# Intrapleural use of urokinase and DNase in pleural infections managed with repeated thoracentesis: A comparative cohort study

**DOI:** 10.1371/journal.pone.0257339

**Published:** 2021-09-21

**Authors:** David Luque Paz, Betsega Bayeh, Pierre Chauvin, Florence Poizeau, Mathieu Lederlin, Mallorie Kerjouan, Charles Lefevre, Bertrand de Latour, Julien Letheulle, Pierre Tattevin, Stéphane Jouneau

**Affiliations:** 1 Department of Respiratory Diseases, Rennes University Hospital, Rennes, France; 2 Infectious Diseases and Intensive Care Unit, Pontchaillou University Hospital, Rennes, France; 3 University of Rennes, Inserm, BRM (Bacterial Regulatory RNAs and Medicine), UMR 1230, Rennes, France; 4 Department of Pneumology and Respiratory Functional Exploration, University Hospital of Tours, Tours, France; 5 EA 7449 (Pharmacoepidemiology and Health Services Research) REPERES, Univ Rennes, CHU Rennes, Rennes, France; 6 PEPS Research Consortium (Pharmacoepidemiology for Health Product Safety), Rennes, France; 7 Department of Dermatology, CHU Rennes, Rennes, France; 8 Department of Radiology, Pontchaillou University Hospital, Rennes, France; 9 Biochemistry Laboratory, Pontchaillou Hospital CHU Rennes, Rennes, France; 10 Department of Thoracic and Cardiovascular Surgery, Rennes University Hospital, Rennes, France; 11 Department of Internal Medicine, Laval Center Hospital, Laval, France; 12 University of Rennes, CHU Rennes, Inserm, EHESP, IRSET (Institut de recherche en santé, environnement et travail)—UMR_S 1085, Rennes, France; Ohio State University Wexner Medical Center Department of Surgery, UNITED STATES

## Abstract

**Introduction:**

Evacuation of infected fluid in pleural infections is essential. To date, the use of an intrapleural fibrinolytic agent such as urokinase and DNase has not yet been assessed in infections managed by repeated therapeutic thoracentesis (RTT).

**Methods:**

We performed a retrospective comparative study of two successive cohorts of consecutive patients with pleural infections from 2001 to 2018. Between 2001 and 2010, patients had RTT with intrapleural urokinase (RTT-U). After 2011, patients received intrapleural urokinase and DNase with RTT (RTT-UD). Data were collected through a standardized questionnaire.

**Results:**

One hundred and thirty-three patients were included: 93 were men and the mean age was 59 years (standard deviation 17.2). Eighty-one patients were treated with a combination of intrapleural urokinase and DNase, and 52 were treated with intrapleural urokinase only. In the RTT-UD, RTT failure occurred in 14 patients (17%) compared to 10 (19%) in the RTT-U group (*P* = 0.82). There was no difference between the two groups in intensive care unit admission, surgical referrals or in-hospital mortality. RTT-UD was associated with faster time to apyrexia (aOR = 0.51, 95%CI [0.37–0.72]), a reduced length of hospital stay (aOR = 0.61, 95%CI [0.52–0.73]) and a higher volume of total pleural fluid retrieved (aOR = 1.38, 95%CI [1.02–1.88]). Complications were rare with only one hemothorax in the RTT-UD group and no pneumothorax requiring drainage in either group.

**Conclusion:**

Compared to urokinase only, intrapleural use of urokinase and DNase in RTT was associated with quicker defervescence, shorter hospital stay and increased volumes of pleural fluid drained. Randomized controlled trials evaluating urokinase and DNase with RTT technique would be required to confirm these results.

## Introduction

Pleural infections are severe infectious diseases, with a mortality rate ranging from 10 to 20% [1, 2]. Incidence of pleural infections has increased in the last few decades and reached 15 cases per 100 000 per year [3–5]. According to guidelines, the two pillars of treatment are effective antibiotics and drainage of the infected liquid [6–8]. Hippocrates already underlined that ‘if an empyema does not rupture, death will occur’. To date, chest tube insertion is the most widespread technique of drainage and is considered as the gold standard treatment in pleural infections. Nevertheless, another strategy is possible, namely repeated therapeutic thoracentesis (RTT). The theoretical advantages of RTT are: i) targeting spaces where loculated collections are the most voluminous; ii) allowing early ambulation; iii) allowing early pleural physiotherapy; iv) avoiding clogging of chest tube. Several studies reported RTT as an effective alternative to chest tube. We previously described a cohort of pleural infections managed by RTT as first-line treatment, with a clinical success rate beyond 80% and low rate of complications related to RTT [9]. In a comparative study, thoracenteses were not inferior to chest tube, with reduced duration of hospitalization and complications rates [10].

Furthermore, releasing fibrinolytic agents into pleural cavity has shown benefits in improving drainage effectiveness in a large randomized controlled trial [11]. In this study, Rahman *et al*. demonstrated that intrapleural use of tissue plasminogen activator (tPA) and deoxyribonuclease (DNase) reduces surgical referral at 3 months and length of hospital stay. In a previous trial, administration of a single fibrinolytic agent–tPA–did not improve any outcome [1]. Intrapleural fibrinolytic therapy has only been assessed once agents have been released through the chest tube and retrieved after one hour. In RTT, fibrinolytic agents and DNase released into the pleural space have a prolonged exposure. Though urokinase or tPa have very short half-lives (30–45 minutes), DNase has prolonged half-life (3–4 hours) increasing its activity on loculations [12, 13]. We aimed to compare outcomes in patients with pleural infections managed by RTT with urokinase and DNase (RTT-UD), versus RTT with urokinase (RTT-U).

## Methods

### Study population

Rennes University Hospital is a 1500-bed tertiary-care hospital, which serves as a referral center in Western France (population catchment area, 1,000,000 inhabitants). We performed a retrospective study of all consecutive patients managed with RTT as first-line treatment for pleural infections in the Rennes University Hospital, France, during years 2001–2018. Non-complicated parapneumonic effusions were excluded from the study. To define pleural infections, we included all adults patients (age ≥ 18 years) with at least one clinical symptom of current lung infection and at least one of the following characteristics for pleural fluid: frank pus (empyema), microorganisms observed after Gram staining, pH <7.2, glucose level <2.2 mmol/L, intrapleural loculations or abundant parapneumonic effusion. We divided our global cohort of patients with pleural infections treated by RTT into two consecutive groups: i) the first from 2001 to 2010, when patients were managed by RTT with adjunction of urokinase (RTT-U) ii) the second from 2011 to 2018, when most patients were managed by RTT with intrapleural use of urokinase and DNase (RTT-UD), following changes in our local protocol.

### Data collection

Medical files of all patients treated by RTT as first-line regimen were reviewed. Data were collected with a standardized questionnaire including demographic, clinical, biological and imaging data. Outcomes were in-hospital mortality, one-year mortality, RTT failure (defined by death related to pleural infection, surgical referral or the need to switch to chest tube drainage), ICU admission, time to apyrexia, length of hospital stay and volume of pleural fluid drained. Apyrexia was defined as an axillary temperature < 38°C during at least 72 hours.

### RTT protocol

The RTT protocol was standardized and was performed at the patient’s bedside under local anesthesia (lidocain 1%) using 8-French laparoscopic trocar (‘Boutin Trocart’ -Novatech®, La Ciotat, France). Intrapleural fibrinolysis protocol was standardized, based on urokinase (Eumedica®, Biarritz, France) 100 000 UI and DNase (Pulmozyme, Roche®) 5000 UI diluted in 50 mL of saline, then instilled by gravity in the pleural space using the pleural trocar at the end of thoracentesis. Patients at risk of hemorrhage or with a bronchopleural fistula were excluded, as these are contraindication for fibrinolysis. Each thoracentesis attempted to evacuate a maximum volume of infected pleural effusion. Thoracentesis were repeated as many times as required, at the discretion of the physician, until sepsis was under control and pleural effusion had significantly decreased. Complications, such as blank thoracentesis, vasovagal reaction or pleural hemorrhage, were collected.

### Statistical analysis

We first described the characteristics of all patients enrolled in the two cohorts. Results were expressed as median and interquartile range (IQR) or mean and standard deviation (SD) for quantitative variables, and relative frequencies for qualitative variables. Missing data for each variable were excluded from the denominator. The Student *t*-test or Mann-Whitney test, and Fisher’s exact test were used, as appropriate. We then performed logistic and linear regression analyses to evaluate the ‘use of urokinase and DNase’. A logarithmic transformation was applied for a non-normally distributed response variable, namely ‘total volume of pleural fluid retrieved’, ‘time to apyrexia after initial pleural drainage’ or ‘hospital length of stay’. A propensity score for treatment allocation was calculated using an adjusted logistic regression model with potential confounders: age, sex, underlying comorbidities (such as COPD, chronic heart failure, diabetes mellitus, malignancy, immunosuppression), severity at admission (based on respiratory failure and sepsis criteria), number of thoracentesis performed, abundance of pleural effusion at admission, positive pleural fluid direct examination, exact RAPID-score (which is a validated prognosis score in pleural infections) and ultrasonography-guided thoracentesis. Thereafter, the overlap weighting method based on propensity score was applied to a logistic regression for the following outcomes: in-hospital mortality, one-year mortality, RTT failure, ICU admission; and linear regression for the following outcomes: length of hospital stay, time to apyrexia, total volume of pleural fluid drained. Covariate balance after overlap weighting was evaluated by checking standardized differences (S1 Fig). Two-tailed p-values were reported, with p<0.05 considered as statistically significant. Statistical analysis was carried out using R-Studio 2015, Integrated Development for R (R-Studio, Boston, MA, USA).

### Ethics

The study was approved by the Ethics Committee of Rennes University Hospital (approval number 12.52). Written informed consent was obtained from all study surviving patients.

## Results

**Fig 1:**
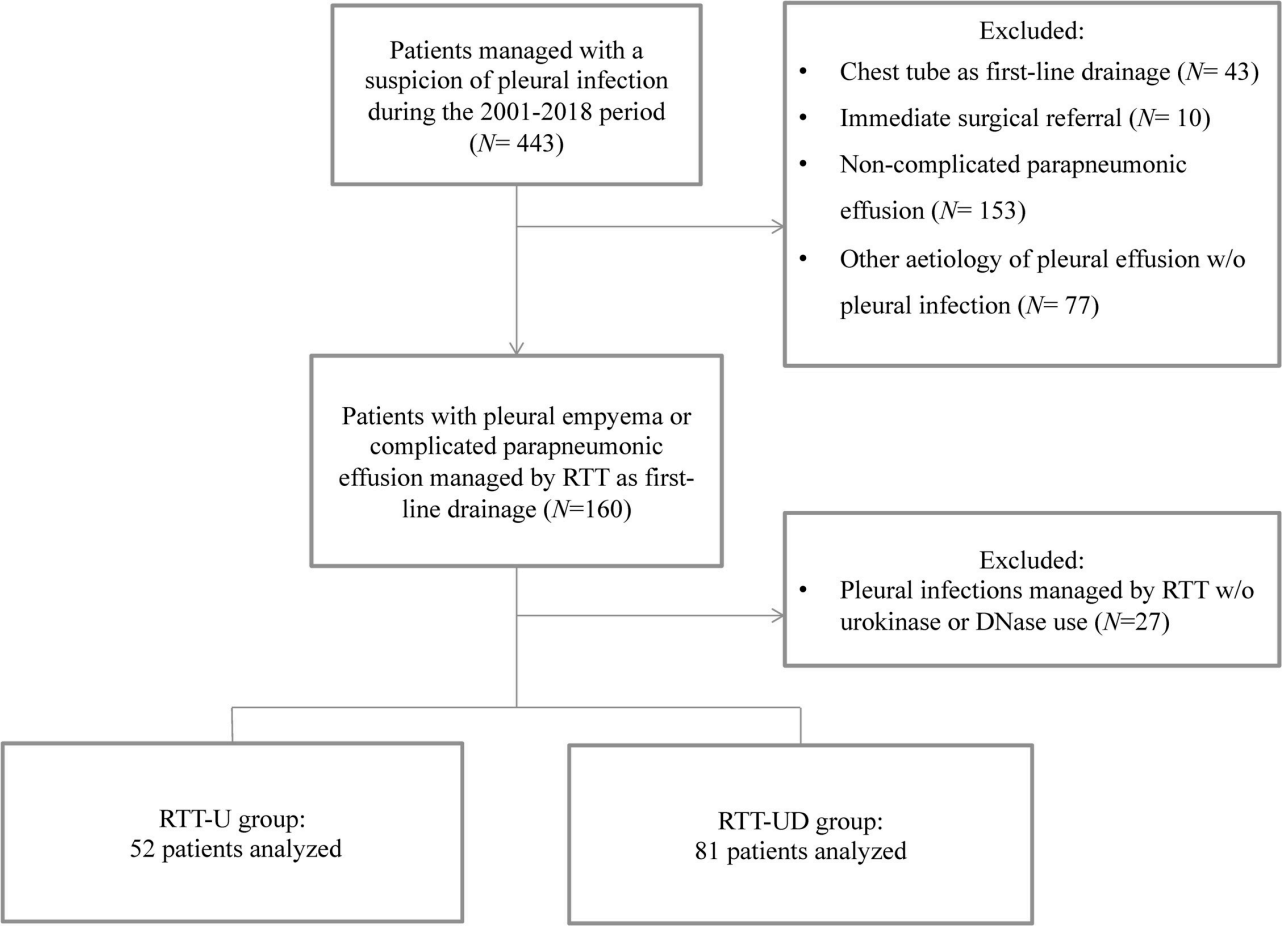
Flowchart of the study. RTT-U: Repeated therapeutic thoracenteses with use of intrapleural urokinase only. RTT-UD: Repeated therapeutic thoracenteses with use of intrapleural urokinase and DNase.

Of the 443 patients with suspected pleural infection, 133 fulfilled criteria for complicated parapneumonic effusion (CPPE) or pleural empyema (PE) (Fig 1). Data on patients from the first group were previously published [9].

The mean age was 59 years (standard deviation, 17.2), 69.2% were men (92/133), 63.9% (85/133) had at least one chronic underlying disease. Infections were mostly community-acquired (116/133, 87.0%). Characteristics of the two groups are summarized in Table 1. Regarding demographical characteristics and comorbidities, the two groups had no significant differences in age, sex-ratio, chronic obstructive pulmonary disease, heart disease, diabetes mellitus or immunodepression. Malignancy was more frequent (n = 7/52 vs. 25/81, *P* = 0.02) in the RTT-UD group, and neurological impairment less frequent (n = 19/52 *vs*. 12/81, *P* = 0.01).

**Table 1:** Baseline characteristics of the study population.

	All, n = 133	RTT-U, n = 52 (%)	RTT-UD, n = 81 (%)	P-value
**Demographical and Clinical characteristics**				
Mean age, years (SD)	59 (17.2)	56 (18.1)	60 (16.4)	0.18
Male gender, n (%)	93 (70%)	36 (69%)	57 (70%)	1
Community-acquired infection, n (%)	116 (87%)	49 (94%)	67 (83%)	0.06
Delay symptoms-admission, days (IQR)	7 (3–15)	9 (4–15)	7 (3–15)	0.06
Smoker, n (%)	81 (61%)	30 (58%)	51 (64%)	0.59
Chronic obstructive pulmonary disease, n (%)	8 (6%)	3 (6%)	5 (7%)	1
Heart disease, n (%)	21 (16%)	7 (13%)	14 (17%)	0.63
Diabetes mellitus, n (%)	22 (17%)	6 (12%)	16 (20%)	0.24
Immunodepression, n (%)	16 (12%)	3 (6%)	13 (16%)	0.10
Alcohol abuse, n (%)	46 (27%)	15 (29%)	21 (27%)	0.84
Malignancy, n (%)	32 (24%)	7 (13%)	25 (31%)	0.02
Chronic liver disease, n (%)	13 (10%)	5 (10%)	8 (10%)	1
Neurological impairment, n (%)	31 (23%)	19 (37%)	12 (15%)	0.01
Non-steroid anti-inflammatory drugs, n (%)	31 (23%)	10 (19%)	21 (30%)	0.21
Corticosteroids, n (%)	11 (8%)	5 (10%)	6 (8%)	0.75
Antibiotics initiated before thoracentesis, n (%)	59 (44%)	23 (44%)	36 (46%)	1
**Radiological data**				
Right side location, n (%)	79 (59%)	33 (63%)	46 (57%)	0.47
Large effusion (> 1/2 thorax), n (%)	57 (43%)	32 (62%)	25 (31%)	0.01
Abundance score (IQR)	3 (3–4)	4 (3–4)	3 (3–3)	0.004
Bilateral effusion, n (%)	10 (8%)	2 (4%)	8 (10%)	0.31
Mediastinal shift, n (%)	30 (23%)	13 (25%)	17 (21%)	0.83
Loculations, n (%)	97 (73%)	31 (60%)	66 (90%)	0.002
**Index of diseases severity**				
Respiratory failure, n (%)	40 (30%)	7 (13%)	33 (41%)	< 0.001
Severe sepsis, n (%)	22 (17%)	3 (6%)	19 (23%)	0.008
Urea, mmol/L (IQR)	6.0 (3.7–8.5)	5.0 (3.35–7.7)	6.0 (4–9.3)	0.11
Albumin, g/L (IQR)	25 (22–31)	22.0 (21.4–23.1)	27 (24.2–32)	<0.001
RAPID-score				
Low risk, n (%)	57 (43%)	26 (50%)	31 (38%)	0.21
Medium risk, n (%)	57 (43%)	18 (35%)	39 (48%)	0.15
High risk, n (%)	19 (14%)	8 (15%)	11 (14%)	0.80
**Pleural fluid analysis**				
Frank pus (empyema), n (%)	83 (62%)	34 (65%)	49 (60%)	0.59
Protein, g/L (IQR)	45 (39.9–49.4)	46 (42–49)	44 (38.9–49.3)	0.44
LDH, IU/L (IQR)	2290 (880–9772)	4141 (1498–17229)	1417 (720.5–5700)	0.003
pH * (IQR)	7.6 (7–8)	7.2 (7.0–7.5)	7.5 (7.5–8)	0.002
Glucose level, mmol/L (IQR)	1 (0.1–4.1)	1 (0.1–4.1)	1 (0.1–3.9)	0.92
Micro-organisms observed on Gram staining, n (%)	53 (40%)	25 (48%)	28 (35%)	0.15
Positive culture, n (%)	56 (42%)	21 (40%)	35 (43%)	0.86
**Microbiological characteristics**				
Positive blood culture, n (%)	18 (14%)	3 (6%)	15 (20%)	0.04
Positive pneumococcal urine antigen, n (%)	15 (10%)	5 (10%)	8 (10%)	1
**Identified bacteria**				
Anaerobic bacteria, n (%)	22 (17%)	10 (19%)	12 (14%)	0.63
*Streptococcus milleri*, n (%)	22 (17%)	8 (15%)	14 (17%)	0.82
*Streptococcus pneumoniae*, n (%)	28 (21%)	9 (17%)	19 (23%)	0.51
Other *Streptococcus* sp., n (%)	12 (9%)	5 (10%)	7 (9%)	1
*Staphylococcus aureus*, n (%)	2 (2%)	1 (2%)	1 (1%)	1
Gram-negative bacteria, n (%)	30 (23%)	11 (21%)	19 (24%)	0.83
*P*. *aeruginosa*, n (%)	3 (2%)	1 (2%)	2 (3%)	1
*Enterobacteriaceae*, n (%)	11 (8%)	1 (2%)	9 (11%)	0.09

* measured by pH indicator strip, method with high variability [40, 41].

IQR = interquartile range.

SD = standard deviation.

In the RTT-UD group, patients had a more severe initial presentation with more respiratory failure at admission (n = 7/52 vs. 33/81, *P*<0.001) and severe sepsis (n = 3/52 vs. 19/81, *P* = 0.008). The risk-groups proportions of the RAPID score, namely low–medium–high, were 50% (n = 26/52), 35% (n = 18/52) and 15% (n = 8/52) in the RTT-U group; whereas proportions in the RTT-UD group were 38.3% (n = 31/81), 48.1% (n = 39/81) and 13.6% (n = 11/81) respectively [2]. Using this score, there were no significant differences in the risk-groups between the two groups.

Biochemical and microbiological parameters of pleural fluid did not differ significantly from one group to the other, except for pleural pH and LDL levels (Table 1). Positive cultures on pleural fluid were found in less than half of cases (n = 56/133, 42.1%). Streptococci were the leading pathogens identified (n = 62/133, 46.6%), followed by Gram-negative bacteria (n = 30/133, 22.6%), strict anaerobic bacteria (n = 22/133, 16.5%), and staphylococci (n = 2/133, 1.5%).

The number of thoracentesis per patient in the two groups were equivalent (4 [3–6] vs. 4 [3–5]; *P* = 0.49) (Table 2). Ultrasonography-guided procedure was more frequent in the recent cohort (54% vs. 84%; *P* = 0.007). Complications of RTT such as iatrogenic pneumothorax (n = 9/133, 6.8%) and vasovagal reaction (4 out of a total of 632 thoracenteses) were low in both groups but almost one third of patients (n = 46/133, 34.6%) had at least one blank thoracentesis during the management of the pleural infection. Antibiotics used did not differ between the two groups, with a large proportion of patients treated by amoxicillin/clavulanic acid combination (58% in RTT-U group *vs*. 60% in RTT-UD group). Amoxicillin alone was prescribed in cases of pleural infection due to *S*. *pneumoniae* or oral streptococci (15% in RTT-U group *vs*. 26% in RTT-UD group). Patients in the RTT-U group received longer antibiotic courses compared to RTT-UD (Table 2).

**Table 2:** Management and outcomes of the study population.

	All (n = 133)	RTT-U (n = 52)	RTT-UD (n = 81)	P-value
**RTT modalities**				
Number of thoracentesis (IQR)	4 (3–6)	4 (3–6)	4 (3–5)	0.49
Duration of management with RTT, days (IQR)	7 (4–11)	8 (4–15)	6 (4–9)	0.008
Delay admission –1st thoracentesis, day (IQR)	1 (0–5)	1 (0–4)	2 (1–5)	0.19
Delay symptoms –1st thoracentesis, days (IQR)	11 (6–18)	12 (7.5–20.5)	11 (6–17)	0.18
Ultrasonography-guided procedure, n (%)	96 (72%)	28 (54%)	68 (84%)	0.007
Total volume of pleural fluid retrieved, mL (IQR)	1225 (500–1888)	1041 (513–1600)	1300 (655–2312.5)	0.17
Blank thoracentesis, n (%)	46 (35%)	14 (27%)	32 (39%)	0.19
**Secondary treatments**				
Chest tube drainage, n (% total patients)	11 (8%)	8 (15%)	3 (4%)	0.02
Surgery, n (% total patients)	11 (8%)	2 (4%)	9 (11%)	0.20
**Other treatments**				
Duration of IV antibiotics, days (IQR)	14 (10–23)	19 (13–32)	12 (7–18.5)	< 0.001
Total duration of antibiotics, days (IQR)	45 (42–50)	47 (43–51)	42 (42–47)	0.03
**Thoracentesis**				
Vasovagal reaction, n (%)	4 (3%)	2 (4%)	2 (2%)	0.64
Iatrogenic pneumothorax, n (%)	9 (7%)	4 (8%)	5 (6%)	0.74
**Outcomes**				
In-hospital death, n (%)	12 (6%)	1 (2%)	7 (9%)	0.25
One-year mortality, n(%)	25 (19%)	9 (17%)	16 (20%)	0.82
Hospital stay, days (IQR)	20 (14–29)	23 (18–41)	16 (12–24)	< 0.001
Fever duration, days (IQR)	8 (4–13)	10 (7–14)	7 (4–11)	0.003
Fever duration after 1st thoracentesis, days (IQR)	6 (3–9)	9 (4–13)	5 (2–8)	< 0.001
RTT failure, n (%)	24 (18%)	10 (19%)	14 (17%)	0.82
ICU admission, n (%)	20 (15%)	8 (15%)	12 (15%)	1
Re-hospitalization rate, n (%)	14 (11%)	6 (12%)	8 (10%)	0.77

RTT = Repeated Therapeutic Thoracentesis.

RTT-U = Repeated Therapeutic Thoracentesis with Urokinase.

RTT-UD = Repeated Therapeutic Thoracentesis with Urokinase and DNase.

ICU = Intensive care unit.

IQR = interquartile range.

Time to apyrexia was significantly shorter in patients treated with the combination of urokinase and DNase (median fever duration: 10 *vs*. 7 days, *P* = 0.003). Fever duration after the first thoracentesis was also reduced in patients treated by RTT-UD (*P*<0.001). In-hospital mortality did not differ between the two groups (*P* = 0.25). Intensive care unit (ICU) admission rates were similar (15% *vs*. 15, *P* = 1).

Among outcomes, there was no difference in RTT failure, in-hospital mortality or one-year mortality between the two groups in the propensity score-weighted analysis (Table 3). RTT failure occurred in 19.2% (n = 10/52) with RTT-U and 17% (n = 14/81) with RTT-UD. Secondary chest tube drainages were less frequent with RTT-UD (4% vs. 15.4%, *P* = 0.02). Patients in the RTT-UD group had a reduced length of hospital stay (16 [12–24] vs. 23 days [18–41], P<0.001) and a higher total volume of pleural fluid retrieved (1041 mL [513–1600] *vs*. 1300 mL [655–2312.5]). However, abundance of pleural effusion was significantly lower in the RTT-UD group using an abundance score ranging from 0 to 5 on initial chest X-ray (*P* = 0.004).

**Table 3:** Results of the overlap propensity score–weighted analysis to estimate the effect of the combination of intrapleural urokinase & DNase.

Outcomes	Intrapleural urokinase + DNase			
	*P-value*	Crude OR (95%CI)	*P-value*	Weighted OR (95%CI)
In-hospital mortality ^a^	0.17	1.06 (0.98–1.14)	0.06	1.07 (0.99–1.15)
One-year mortality ^a^	0.72	1.02 (0.89–1.18)	0.73	1.02 (0.90–1.16)
RTT failure ^a^	0.78	0.98 (0.86–1.12)	0.25	1.08 (0.95–1.23)
ICU admission ^a^	0.93	0.99 (0.87–1.13)	0.87	0.99 (0.90–1.10)
Time to apyrexia ^b^, days	< 0.001	0.54 (0.38–0.76)	< 0.001	0.51 (0.37–0.72)
Length of hospital stay ^b^, days	< 0.001	0.62 (0.51–0.75)	< 0.001	0.61 (0.52–0.73)
Volume of pleural fluid drained ^b^, mL	0.15	1.27 (0.91–1.77)	0.04	1.38 (1.02–1.88)

^a^ Logistic regression model used.

^b^ Linear regression model used.

OR = Odds Ratio.

RTT = Repeated Therapeutic Thoracentesis.

ICU = Intensive Care Unit.

Estimates of weighted odds-ratios (wORs) obtained with the overlap weighting method based on the propensity score are reported in Table 3. Time to apyrexia (wOR = 0.51, 95%CI [0.37–0.72]) and the length of hospital stay (wOR = 0.61, 95%CI [0.52–0.73]) were significantly reduced in the RTT-UD group. The total volume of pleural fluid retrieved was significantly and independently higher with the use of urokinase and DNase (wOR = 1.38, 95%CI [1.02–1.88]). Other outcomes, namely in-hospital mortality, one-year mortality, RTT failure and ICU admission did not differ from one group to the other after adjusted analysis.

Non-purulent pleural fluid is associated with poorer prognosis than pleural infections with purulent fluid in the RAPID-score. In the subgroup of patients with non-purulent pleural fluid, only one RTT failure (n = 1/27) was reported in the RTT-U group and no RTT failure (n = 0/32) was encountered in the RTT-UD group. Event-free survivals according to the purulence of pleural fluid are plotted in Kaplan-Meier curves (Supplementary data, S1 Fig).

## Discussion

Intrapleural fibrinolytic and DNase agents in pleural infections have been assessed only on patients drained with chest tube. To our knowledge, this study is the first to evaluate intrapleural fibrinolytic agent–urokinase—and DNase in pleural infections managed by RTT. Our work underlines many important messages: i) Time to apyrexia, length of hospital stay and the total volume of pleural fluid retrieved were optimized with a combination of intrapleural urokinase and DNase, ii) Mortality rates, RTT failures and surgical referral were low and did not differ between the two groups, iii) RTT is safe, with a low rate of iatrogenic complications, iv) in patients treated with RTT-UD who had non-purulent pleural fluid, no failure was reported.

In our center, we opted for urokinase instead of streptokinase or alteplase because it seems that urokinase is associated with a lower incidence of bleeding or allergic reaction [12, 14–16]. Moreover, two randomized placebo-controlled trials found that urokinase decreased duration of hospitalization, with faster defervescence and improved pleural drainage [17–19]. In addition, a meta-analysis supported that urokinase use could reduce surgical intervention (OR = 0.33; 95%CI: 0.14–0.78) [20]. Adding DNase is based on clear-cut benefits demonstrated in the MIST-2 trial [21, 22]. It has also been proven that this treatment strategy is highly cost-effective [23]. Moreover, a combination including DNase was more effective in experimental studies, with a reduction of pleural fluid viscosity and an increased volume of pleural fluid drained [24, 25]. These are the reasons why we used this combination with RTT. In line with previous studies, we found that the total volume of pleural fluid drained was higher with intrapleural urokinase and DNase, even though pleural effusion seemed to be less abundant and more loculated in the RTT-UD group. Of note, loculations were more commonly detected in the more recent group, probably explained by development of pleural ultrasonography in our department. On the other hand, we have no explanation for the fact that abundance of pleural effusion on chest imaging was lower in the RTT-UD group. Our work supports an enhanced effective drainage with the use of urokinase and DNase in pleural infections managed by RTT. In addition, time to apyrexia has been assessed as an outcome parameter in infectious diseases, where inappropriate therapies were associated with longer duration of fever [26, 27]. Moreover, several studies identified prolonged fever as a factor of poor outcome [28–30]. In this study, defervescence was lower in the RTT-UD group, arguing for its determinant role in controlling pleural infections.

In a landmark randomized controlled trial, Rahman *et al*. showed a reduction in hospital stay and a decrease in surgical referral with the use of t-PA and DNase in patients treated with chest tube. Herein, we did not find differences in mortality or surgical referral, but our study may have been underpowered due to limited sample size. The absence of difference in mortality rates comes as no surprise since no evidence of reduction in overall mortality has ever been proven [15]. In a meta-analysis, Janda and Swiston. brought to light that there was no difference in mortality rates with intrapleural fibrinolytic therapy [31]. In this work, we reported mortality and surgical referral rates concordant with other studies in literature, mostly using chest tube drainage strategy [32].

RTT had previously been identified as an effective alternative to chest tube in complicated parapneumonic effusion [9]. An experimental study focused on thoracentesis in the treatment of pleural empyema in rabbits revealed that thoracentesis was at least as effective as chest tube in terms of mortality [33]. In a recent review of literature, only few and old studies of limited sample size reported low rates of success with this strategy of drainage [34]. In other studies, clinical success rates ranged from 81 to 100% [9, 10, 35, 36]. To our knowledge, our study is the largest to date which assessed RTT in pleural infection management. We reported clinical success in 82% of cases, and only three patients required chest tube as second-line treatment in the RTT-UD group. Additionally, thoracentesis with adjunctive fibrinolytic and DNase could be a strategy avoiding more invasive procedures [37, 38]. Considering the half-life of DNase of 3–4 hours, a greater exposition time may improve effects on loculations [13]. In our study, even if fibrinolytic agents (i.e urokinase) and DNase remained in the pleural space until the next thoracentesis, adverse effects were rare with only one hemothorax and no pneumothorax requiring drainage.

Ferguson *et al*. found that pleural empyema was associated with failure of RTT [39]. However, in the RAPID-score, non-purulent pleural fluid was a predictor of poor outcome. We reported no failure in patients with non-purulent pleural fluid treated by RTT-UD. It suggests that RTT-UD could be considered as an effective alternative treatment strategy of pleural infections with non-purulent fluid. In case of empyema, switching to chest tube or surgery should be considered if necessary.

Our work has limitations. First, as a single-center study, our findings may differ from others. Second, as data were collected retrospectively over a large period of time (2001–2018), this study has potential biases. Nonetheless, heterogeneity in practice between the two groups was limited to adjunction of intrapleural DNase after 2011 and increased use of pleural ultrasonography. In addition, there were few missing data in the final database. Third, this study focused solely on pleural infections managed by RTT as first-line treatment. Hence, our population did not enroll most patients with severe presentation, such as patients admitted straight to ICU–where chest tube is first-line treatment- or patients who required early surgical referral. Fourth, the study was not designed to compare outcomes between patients receiving intrapleural fibrinolytic agent plus DNase through chest tube and by RTT. Lastly, this study did not assess the impact of the main partner for treatment success: antimicrobial treatment. However, our practices regarding antibiotic use for pleural infections did not significantly change during the study period.

In conclusion, intrapleural use of urokinase and DNase was associated with better outcomes than urokinase only in the management of pleural infections managed with RTT. Pleural infections are hard-to-treat infections, but RTT failures were infrequent, confirming the efficacy of RTT-UD as an alternative strategy to chest tube drainage, especially in complicated parapneumonic effusions.

## Supporting information

S1 Fig Covariate balance of absolute standardized differences before and after overlap propensity score weighting comparing values between patients in RTT-U and RTT-UD groups. PS: Propensity-Score, RTT: Repeated Therapeutic Thoracentesis, COPD: Chronic Obstructive Pulmonary Disease.
(TIF)

S2 Fig Event free survival in pleural infections managed with RTT according to the purulence of the pleural fluid drained. (TIF)

S1 Data (XLSX)

## References

[1] Maskell NA, Davies CWH, Nunn AJ, Hedley EL, Gleeson FV, Miller R, et al. U.K. Controlled Trial of Intrapleural Streptokinase for Pleural Infection. New England Journal of Medicine. 2005 Mar 3;352(9):865–74. doi: 10.1056/NEJMoa042473 15745977

[2] Rahman NM, Kahan BC, Miller RF, Gleeson FV, Nunn AJ, Maskell NA. A Clinical Score (RAPID) to Identify Those at Risk for Poor Outcome at Presentation in Patients With Pleural Infection. Chest. 2014 Apr;145(4):848–55. doi: 10.1378/chest.13-1558 24264558

[3] Grijalva CG, Zhu Y, Nuorti JP, Griffin MR. Emergence of parapneumonic empyema in the USA. Thorax. 2011 Aug 1;66(8):663–8. doi: 10.1136/thx.2010.156406 21617169 PMC4820002

[4] Lehtomäki A, Nevalainen R, Ukkonen M, Nieminen J, Laurikka J, Khan J. Trends in the Incidence, Etiology, Treatment, and Outcomes of Pleural Infections in Adults Over a Decade in a Finnish University Hospital. Scandinavian Journal of Surgery. 2019 Feb 21;145749691983214. 10.1177/1457496919832146 30791827

[5] Finley C, Clifton J, FitzGerald JM, Yee J. Empyema: An increasing concern in Canada. Can Respir J. 2008 Mar;15(2):85–9. doi: 10.1155/2008/975312 18354748 PMC2677840

[6] Davies HE, Davies RJO, Davies CWH. Management of pleural infection in adults: British Thoracic Society pleural disease guideline 2010. Thorax. 2010 Aug 1;65(Suppl 2):ii41–53. doi: 10.1136/thx.2010.137000 20696693

[7] Colice GL, Curtis A, Deslauriers J, Heffner J, Light R, Littenberg B, et al. Medical and surgical treatment of parapneumonic effusions : an evidence-based guideline. Chest. 2000 Oct;118(4):1158–71. doi: 10.1378/chest.118.4.1158 11035692

[8] Shen KR, Bribriesco A, Crabtree T, Denlinger C, Eby J, Eiken P, et al. The American Association for Thoracic Surgery consensus guidelines for the management of empyema. The Journal of Thoracic and Cardiovascular Surgery. 2017 Jun 1;153(6):e129–46. doi: 10.1016/j.jtcvs.2017.01.030 28274565

[9] Letheulle J, Tattevin P, Saunders L, Kerjouan M, Léna H, Desrues B, et al. Iterative thoracentesis as first-line treatment of complicated parapneumonic effusion. PLoS ONE. 2014;9(1):e84788. doi: 10.1371/journal.pone.0084788 24400113 PMC3882258

[10] Storm HK, Krasnik M, Bang K, Frimodt-Møller N. Treatment of pleural empyema secondary to pneumonia: thoracocentesis regimen versus tube drainage. Thorax. 1992 Oct;47(10):821–4. doi: 10.1136/thx.47.10.821 1481185 PMC464066

[11] Rahman NM, Maskell NA, West A, Teoh R, Arnold A, Mackinlay C, et al. Intrapleural Use of Tissue Plasminogen Activator and DNase in Pleural Infection. New England Journal of Medicine. 2011 Aug 11;365(6):518–26. doi: 10.1056/NEJMoa1012740 21830966

[12] Bouros D, Tzouvelekis A, Antoniou KM, Heffner JE. Intrapleural fibrinolytic therapy for pleural infection. Pulm Pharmacol Ther. 2007;20(6):616–26. doi: 10.1016/j.pupt.2006.08.001 17049447

[13] Piccolo F, Popowicz N, Wong D, Lee YCG. Intrapleural tissue plasminogen activator and deoxyribonuclease therapy for pleural infection. J Thorac Dis. 2015 Jun;7(6):999–1008. doi: 10.3978/j.issn.2072-1439.2015.01.30 26150913 PMC4466425

[14] Alemán C, Porcel JM, Alegre J, Ruiz E, Bielsa S, Andreu J, et al. Intrapleural Fibrinolysis with Urokinase Versus Alteplase in Complicated Parapneumonic Pleural Effusions and Empyemas: A Prospective Randomized Study. Lung. 2015 Dec;193(6):993–1000. doi: 10.1007/s00408-015-9807-6 26423784

[15] Altmann ES, Crossingham I, Wilson S, Davies HR. Intra-pleural fibrinolytic therapy versus placebo, or a different fibrinolytic agent, in the treatment of adult parapneumonic effusions and empyema. Cochrane Database Syst Rev. 2019 Oct 30;2019(10). doi: 10.1002/14651858.CD002312.pub4 31684683 PMC6819355

[16] Abu-Daff S, Maziak DE, Alshehab D, Threader J, Ivanovic J, Deslaurier V, et al. Intrapleural fibrinolytic therapy (IPFT) in loculated pleural effusions—analysis of predictors for failure of therapy and bleeding: a cohort study. BMJ Open. 2013;3(2). doi: 10.1136/bmjopen-2012-001887 23377992 PMC3586180

[17] Bouros D, Schiza S, Patsourakis G, Chalkiadakis G, Panagou P, Siafakas NM. Intrapleural streptokinase versus urokinase in the treatment of complicated parapneumonic effusions: a prospective, double-blind study. Am J Respir Crit Care Med. 1997 Jan 1;155(1):291–5. doi: 10.1164/ajrccm.155.1.9001327 9001327

[18] Tuncozgur B, Ustunsoy H, Sivrikoz MC, Dikensoy O, Topal M, Sanli M, et al. Intrapleural urokinase in the management of parapneumonic empyema: a randomised controlled trial. Int J Clin Pract. 2001 Dec;55(10):658–60. 11777287

[19] Majid A, Kheir F, Folch A, Fernandez-Bussy S, Chatterji S, Maskey A, et al. Concurrent Intrapleural Instillation of Tissue Plasminogen Activator and DNase for Pleural Infection. A Single-Center Experience. Ann Am Thorac Soc. 2016 Sep;13(9):1512–8. doi: 10.1513/AnnalsATS.201602-127OC 27333122

[20] Nie W, Liu Y, Ye J, Shi L, Shao F, Ying K, et al. Efficacy of intrapleural instillation of fibrinolytics for treating pleural empyema and parapneumonic effusion: a meta-analysis of randomized control trials. Clin Respir J. 2014 Jul;8(3):281–91. doi: 10.1111/crj.12068 24428897

[21] Rahman NM. Intrapleural agents for pleural infection: fibrinolytics and beyond. Curr Opin Pulm Med. 2012 Jul;18(4):326–32. doi: 10.1097/MCP.0b013e3283531149 22487944

[22] Chaddha U, Agrawal A, Feller-Kopman D, Kaul V, Shojaee S, Maldonado F, et al. Use of fibrinolytics and deoxyribonuclease in adult patients with pleural empyema: a consensus statement. Lancet Respir Med. 2021 Feb 2; doi: 10.1016/S2213-2600(20)30533-6 33545086

[23] Luengo-Fernandez R, Penz E, Dobson M, Psallidas I, Nunn AJ, Maskell NA, et al. Cost-effectiveness of intrapleural use of tissue plasminogen activator and DNase in pleural infection: evidence from the MIST2 randomised controlled trial. Eur Respir J. 2019 Aug;54(2). doi: 10.1183/13993003.01550-2018 31097519

[24] Gehlen M, Fraga JC, Amantea SL, Silveira NP, Kulczynski J, Roesch E, et al. Alteplase and DNase for the treatment of pleural empyema in rats. Pulmonary Pharmacology & Therapeutics. 2019 Apr;55:1–4. doi: 10.1016/j.pupt.2019.01.001 30648619

[25] Zhu Z, Hawthorne ML, Guo Y, Drake W, Bilaceroglu S, Misra HL, et al. Tissue Plasminogen Activator Combined With Human Recombinant Deoxyribonuclease Is Effective Therapy for Empyema in a Rabbit Model. Chest. 2006 Jun;129(6):1577–83. doi: 10.1378/chest.129.6.1577 16778278

[26] Kim S-H, Park W-B, Lee C-S, Kang C-I, Bang J-W, Kim H-B, et al. Outcome of inappropriate empirical antibiotic therapy in patients with Staphylococcus aureus bacteraemia: analytical strategy using propensity scores. Clinical Microbiology and Infection. 2006 Jan 1;12(1):13–21. doi: 10.1111/j.1469-0691.2005.01294.x 16460541

[27] Lee C-C, Wang J-L, Lee C-H, Hsieh C-C, Hung Y-P, Hong M-Y, et al. Clinical Benefit of Appropriate Empirical Fluoroquinolone Therapy for Adults with Community-Onset Bacteremia in Comparison with Third-Generation-Cephalosporin Therapy. Antimicrob Agents Chemother [Internet]. 2017 Jan 24 [cited 2021 Feb 3];61(2). Available from: https://www.ncbi.nlm.nih.gov/pmc/articles/PMC5278695/ doi: 10.1128/AAC.02174-16 27855072 PMC5278695

[28] Circiumaru B, Baldock G, Cohen J. A prospective study of fever in the intensive care unit. Intensive Care Medicine. 1999 Jul 22;25(7):668–73. doi: 10.1007/s001340050928 10470569

[29] Laupland KB, Shahpori R, Kirkpatrick AW, Ross T, Gregson DB, Stelfox HT. Occurrence and outcome of fever in critically ill adults*: Critical Care Medicine. 2008 May;36(5):1531–5. doi: 10.1097/CCM.0b013e318170efd3 18434882

[30] Rehman T, deBoisblanc BP. Persistent Fever in the ICU. Chest. 2014 Jan;145(1):158–65. doi: 10.1378/chest.12-2843 24394828

[31] Janda S, Swiston J. Intrapleural fibrinolytic therapy for treatment of adult parapneumonic effusions and empyemas: a systematic review and meta-analysis. Chest. 2012 Aug;142(2):401–11. doi: 10.1378/chest.11-3071 22459772

[32] Ferreiro L, Porcel JM, Bielsa S, Toubes ME, Álvarez-Dobaño JM, Valdés L. Management of pleural infections. Expert Review of Respiratory Medicine. 2018 Jun 3;12(6):521–35. doi: 10.1080/17476348.2018.1475234 29781330

[33] Sasse S, Nguyen T, Teixeira LR, Light R. The Utility of Daily Therapeutic Thoracentesis for the Treatment of Early Empyema. Chest. 1999 Dec;116(6):1703–8. doi: 10.1378/chest.116.6.1703 10593798

[34] Jouneau S, Letheulle J, Desrues B. Repeated therapeutic thoracentesis to manage complicated parapneumonic effusions. Curr Opin Pulm Med. 2015 Jul;21(4):387–92. doi: 10.1097/MCP.0000000000000171 26016584

[35] Mandal AK, Thadepalli H. Treatment of spontaneous bacterial empyema thoracis. J Thorac Cardiovasc Surg. 1987 Sep;94(3):414–8. 3626603

[36] Simmers TA, Jie C, Sie B. Minimally invasive treatment of thoracic empyema. Thorac Cardiovasc Surg. 1999 Apr;47(2):77–81. doi: 10.1055/s-2007-1013115 10363605

[37] Kheir F, Thakore S, Mehta H, Jantz M, Parikh M, Chee A, et al. Intrapleural Fibrinolytic Therapy versus Early Medical Thoracoscopy for Treatment of Pleural Infection. Randomized Controlled Clinical Trial. Ann Am Thorac Soc. 2020 Aug;17(8):958–64. doi: 10.1513/AnnalsATS.202001-076OC 32421353

[38] Idell S, Rahman NM. Intrapleural Fibrinolytic Therapy for Empyema and Pleural Loculation: Knowns and Unknowns. Ann Am Thorac Soc. 2018 May;15(5):515–7. doi: 10.1513/AnnalsATS.201711-848PS 29361235 PMC5955053

[39] Ferguson AD, Prescott RJ, Selkon JB, Watson D, Swinburn CR. The clinical course and management of thoracic empyema. QJM. 1996 Apr 1;89(4):285–90. doi: 10.1093/qjmed/89.4.285 8733515

[40] Lesho EP, Roth BJ. Is pH Paper an Acceptable, Low-Cost Alternative to the Blood Gas Analyzer for Determining Pleural Fluid pH? Chest. 1997 Nov;112(5):1291–2. doi: 10.1378/chest.112.5.1291 9367470

[41] Ng L, Dabscheck E, Hew M. Diagnosis of complicated parapneumonic effusion by pleural pH measurement is jeopardized by inadequate physician knowledge and guideline-discordant laboratory practice. Respiratory Medicine. 2017 Jan;122:30–2. doi: 10.1016/j.rmed.2016.11.012 27993288

