# Supplementary figures and images for: Intrapleural use of urokinase and DNase in pleural infections managed with repeated thoracentesis: A comparative cohort study
Source: PLoS One. 2021 Sep 21;16(9):e0257339. doi: 10.1371/journal.pone.0257339 (PMC8454966; doi:10.1371/journal.pone.0257339)

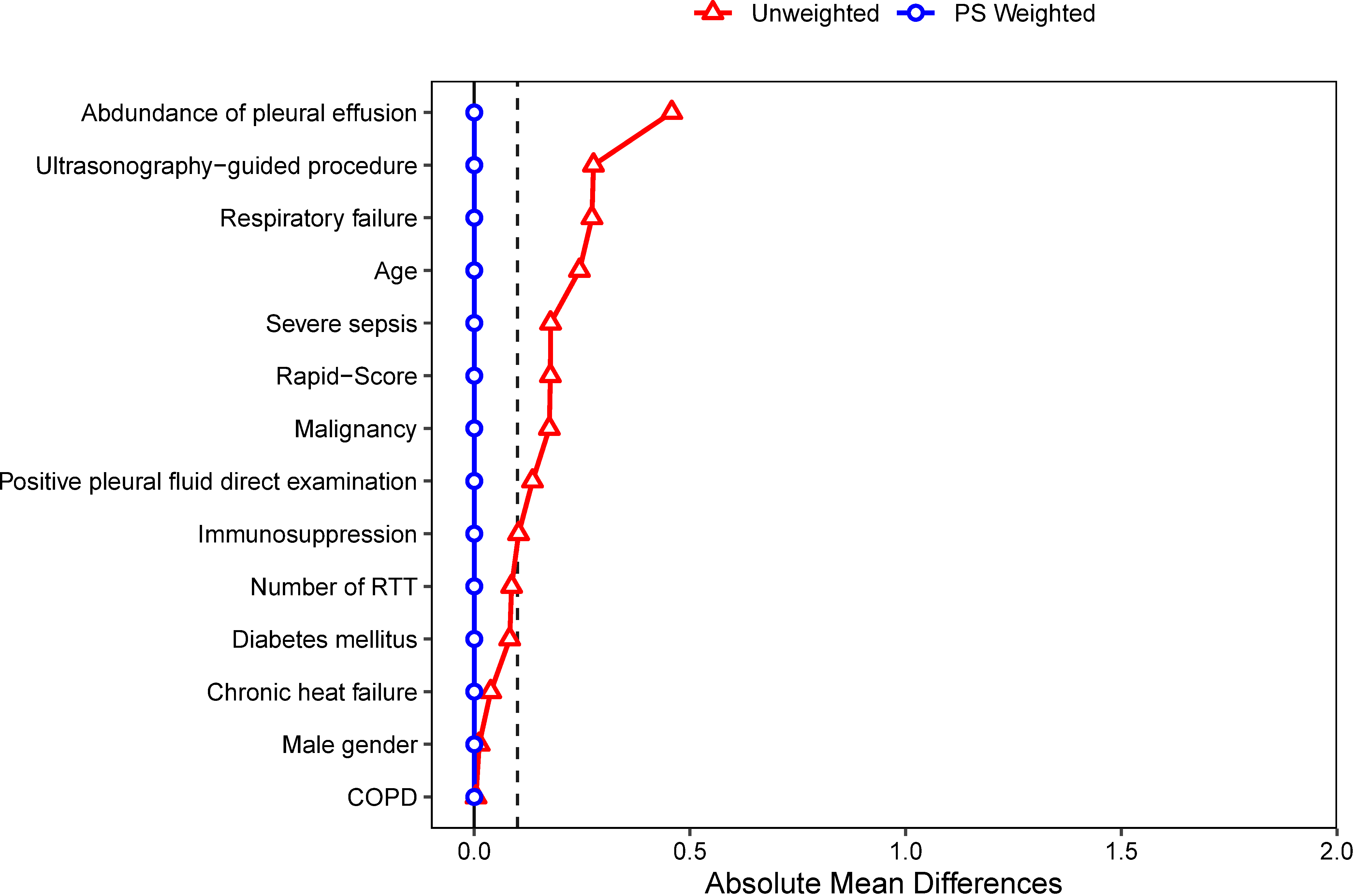

Supplement: S1 Fig — PS: Propensity-Score, RTT: Repeated Therapeutic Thoracentesis, COPD: Chronic Obstructive Pulmonary Disease. (TIF) [file pone.0257339.s001.tif]

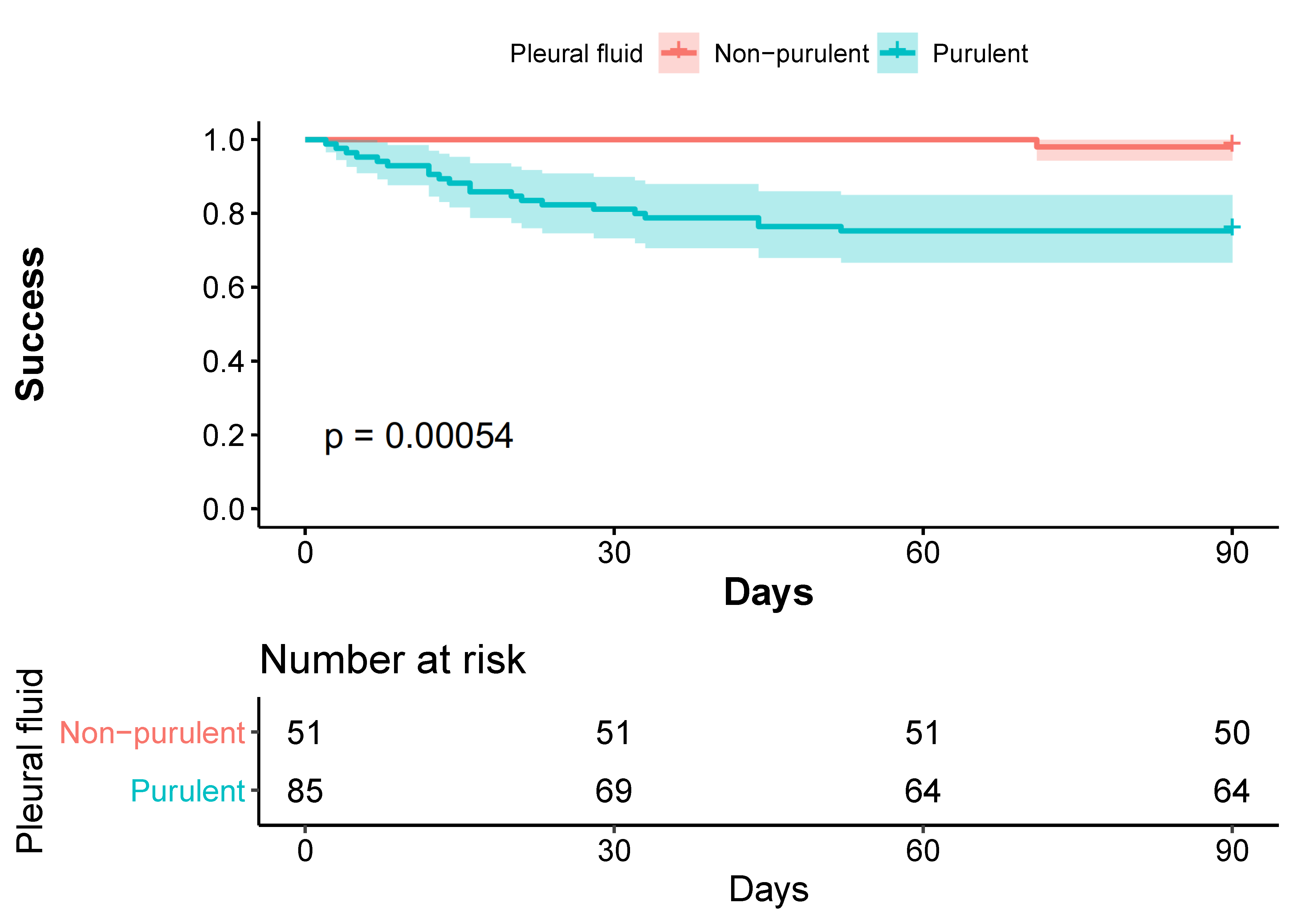

Supplement: S2 Fig — (TIF) [file pone.0257339.s002.tif]
